# Development of a severity of disease score and classification model by machine learning for hospitalized COVID-19 patients

**DOI:** 10.1371/journal.pone.0240200

**Published:** 2021-04-21

**Authors:** Miguel Marcos, Moncef Belhassen-García, Antonio Sánchez-Puente, Jesús Sampedro-Gomez, Raúl Azibeiro, Pedro-Ignacio Dorado-Díaz, Edgar Marcano-Millán, Carolina García-Vidal, María-Teresa Moreiro-Barroso, Noelia Cubino-Bóveda, María-Luisa Pérez-García, Beatriz Rodríguez-Alonso, Daniel Encinas-Sánchez, Sonia Peña-Balbuena, Eduardo Sobejano-Fuertes, Sandra Inés, Cristina Carbonell, Miriam López-Parra, Fernanda Andrade-Meira, Amparo López-Bernús, Catalina Lorenzo, Adela Carpio, David Polo-San-Ricardo, Miguel-Vicente Sánchez-Hernández, Rafael Borrás, Víctor Sagredo-Meneses, Pedro-Luis Sanchez, Alex Soriano, José-Ángel Martín-Oterino

**Affiliations:** 1 Department of Internal Medicine, University Hospital of Salamanca-IBSAL, University of Salamanca, Salamanca, Spain; 2 Department of Cardiology, University Hospital of Salamanca-IBSAL, University of Salamanca, Salamanca, Spain; 3 CIBERCV, Instituto de Salud Carlos III, Madrid, Spain; 4 Department of Hematology, University Hospital of Salamanca-IBSAL, University of Salamanca, Salamanca, Spain; 5 Department of Intensive Care Medicine, University Hospital of Salamanca-IBSAL, University of Salamanca, Salamanca, Spain; 6 Department of Infectious Diseases, Hospital Clínic-Universitat de Barcelona, IDIBAPS, Barcelona, Spain; 7 Department of Anesthesiology and Reanimation, University Hospital of Salamanca-IBSAL, University of Salamanca, Salamanca, Spain; 8 Department of Emergency Medicine, University Hospital of Salamanca-IBSAL, University of Salamanca, Salamanca, Spain; Technion - Israel Institute of Technology, ISRAEL

## Abstract

**Background:**

Efficient and early triage of hospitalized Covid-19 patients to detect those with higher risk of severe disease is essential for appropriate case management.

**Methods:**

We trained, validated, and externally tested a machine-learning model to early identify patients who will die or require mechanical ventilation during hospitalization from clinical and laboratory features obtained at admission. A development cohort with 918 Covid-19 patients was used for training and internal validation, and 352 patients from another hospital were used for external testing. Performance of the model was evaluated by calculating the area under the receiver-operating-characteristic curve (AUC), sensitivity and specificity.

**Results:**

A total of 363 of 918 (39.5%) and 128 of 352 (36.4%) Covid-19 patients from the development and external testing cohort, respectively, required mechanical ventilation or died during hospitalization. In the development cohort, the model obtained an AUC of 0.85 (95% confidence interval [CI], 0.82 to 0.87) for predicting severity of disease progression. Variables ranked according to their contribution to the model were the peripheral blood oxygen saturation (SpO2)/fraction of inspired oxygen (FiO2) ratio, age, estimated glomerular filtration rate, procalcitonin, C-reactive protein, updated Charlson comorbidity index and lymphocytes. In the external testing cohort, the model performed an AUC of 0.83 (95% CI, 0.81 to 0.85). This model is deployed in an open source calculator, in which Covid-19 patients at admission are individually stratified as being at high or non-high risk for severe disease progression.

**Conclusions:**

This machine-learning model, applied at hospital admission, predicts risk of severe disease progression in Covid-19 patients.

## Introduction

Since late 2019, a pneumonia outbreak caused by coronavirus SARS-CoV-2 began in the Chinese city of Wuhan and has evolved into a global pandemic [1]. Clinical manifestations of patients with SARS-CoV-2 infection range from mild disease (e.g., only fever or cough) to critically ill cases with acute respiratory distress syndrome and septic shock. In a large report from the Chinese Center for Disease Control and Prevention, with 44415 cases, 36160 (81%) were described as mild, 6168 (14%) as severe, and 2087 (5%) as critical illness, with a mortality of 49% in the latter group [2]. Due to this variability, several factors have been identified to predict increased severity, such as older age, neutrophilia, organ dysfunction, coagulopathy, or elevated D-dimer levels [3].

Machine-learning is a subfield of computer science and statistics that has received growing interest in medicine, especially in infectious diseases, and has allowed to develop tools to predict clinical outcomes such as the occurrence of sepsis in intensive care units or the diagnosis of surgical site infection [4]. Therefore, in this context of worldwide health emergency, early detection of patients who are likely to develop critical illness is of paramount importance and may aid in delivering proper care and optimizing use of limited intensive care resources.

For this purpose, we report here a machine-learning model able to predict risk of severity of disease progression in Covid-19 patients at the time of admission, developed and validated in two large cohorts of patients from two university hospitals, including easy-to-collect variables such as peripheral blood oxygen saturation (SpO2)/fraction of inspired oxygen (FiO2) ratio, age, estimated glomerular filtration rate, procalcitonin, C-reactive protein, updated Charlson comorbidity index and lymphocytes.

## Material and methods

### Study design and data sources

We conducted a training, validation an external-testing study on an intelligence-based machine-learning model [5], using clinical and laboratory features obtained at hospital admission. A data set from 918 confirmed Covid-19 patients from the University Hospital of Salamanca, Spain, was used for training and internal validation. For external testing we included 352 Covid-19 patients from another university hospital (Hospital Clinic of Barcelona, Spain). A flowchart illustrating the detailed steps involved in our score development is provided as S1 Fig.

Institutional approval was provided by the Ethics Committee of the University Hospital of Salamanca (2020/03/470) and the Comité Ètic d’Investigació Clínica of the Hospital Clínic of Barcelona (HCB/2020/0273), which waived the need for informed consent. All data set were anonymously analyzed, and the study was performed following current recommendation of the Declaration of Helsinki [6].

### Task definition

The aim of our study was to develop and validate a machine-learning model to predict, at the moment of hospital admission, the likelihood that a Covid-19 patient will die or require invasive mechanical ventilation during hospitalization. A secondary objective was to deploy this model into a simple clinical digital application to facilitate its use in real time.

Input data (features) consists in demographic variables (including age and sex), individual comorbidities and Charlson Comorbidity Index, chronic medical treatment, clinical characteristics, physical examination parameters, and biochemical parameters available at hospital admission (Tables 1 and 2). As for the corresponding outcome (label), we defined severity of disease progression during hospitalization as the use of mechanical ventilation or death.

**Table 1:** Admission characteristics of patients from internal validation cohort by outcome.

		Severity of disease progression		
Characteristics	Total (n = 918)	Non-severe (n = 555)	Severe (n = 363)	*P*-Value
Age, years (mean, [SD])	72.8 (14.5)	68.6 (14.7)	79.2 (11.5)	<0.001
Male, n (%)	531 (57.8%)	310 (55.9%)	221 (60.9%)	0.133
Community-acquired infection (n = 887)	822 (92.7%)	480 (90.7%)	342 (95.5%)	0.008
**Comorbidity**				
Updated Charlson comorbidity index, mean (SD) (n = 915)	1.2 (1.7)	0.9 (1.6)	1.8 (1.8)	<0.001
Classic Charlson comorbidity index, mean (SD) (n = 915)	1.5 (1.8)	1.2 (1.7)	2.0 (1.9)	<0.001
Myocardial infarction, n (%)	104 (11.3%)	50 (9.0%)	54 (14.9%)	0.008
Congestive heart failure, n (%)	129 (14.1%)	55 (9.9%)	74 (20.4%)	<0.001
Peripheral vascular disease, n (%)	30 (3.3%)	15 (2.7%)	15 (4.1%)	0.257
Arrhythmia, n (%)	131 (14.3%)	61 (11.0%)	70 (19.4%)	<0.001
Hypertension, n (%)	489 (53.3%)	263 (47.4%)	226 (62.4%)	<0.001
Cerebrovascular accident, n (%)	91 (9.9%)	34 (6.1%)	57 (15.7%)	<0.001
Dementia, n (%)	138 (15.0%)	55 (9.9%)	83 (22.9%)	<0.001
Other central nervous system diseases, n (%)	77 (8.4%)	38 (6.8%)	39 (10.8%)	0.039
Hemiplegia, n (%)	18 (2.0%)	6 (1.1%)	12 (3.3%)	0.026
Current smoking, n (%)	56 (6.8%)	38 (7.6%)	18 (5.4%)	0.259
Former/current smoking, n (%)	196 (23.6%)	107 (21.5%)	89 (26.9%)	0.080
Chronic obstructive pulmonary disease, n (%)	66 (7.2%)	29 (5.2%)	37 (10.2%)	0.006
Asthma, n (%)	0.1 (0.2)	0.1 (0.3)	0.0 (0.2)	0.078
Other chronic pulmonary disease, n (%)	49 (5.3%)	27 (4.9%)	22 (6.1%)	0.454
Rheumatological disorder, n (%)	56 (6.1%)	25 (4.5%)	31 (8.6%)	0.016
Intestinal inflammatory disease, n (%)	49 (5.3%)	27 (4.9%)	22 (6.1%)	0.455
Peptic ulcer disease, n (%)	23 (2.5%)	16 (2.9%)	7 (1.9%)	0.398
Chronic kidney disease (eGFR<30), n (%)	50 (5.5%)	15 (2.7%)	35 (9.7%)	<0.001
Obesity, n (%)	192 (25.2%)	126 (26.9%)	66 (22.4%)	0.198
Diabetes, n (%)	213 (23.2%)	120 (21.6%)	93 (25.6%)	0.174
Dyslipidemia, n (%)	351 (38.4%)	204 (36.8%)	147 (40.8%)	0.237
Other endocrine disease, n (%)	112 (12.2%)	66 (11.9%)	46 (12.7%)	0.757
Malignancy, n (%)	130 (14.2%)	60 (10.8%)	70 (19.3%)	<0.001
Solid tumor	109 (11.9%)	51 (9.2%)	58 (16.0%)	0.002
Leukemia	9 (1.0%)	6 (1.1%)	3 (0.8%)	1.000
Lymphoma	13 (1.4%)	3 (0.5%)	10 (2.8%)	0.008
Trasplant recipient, n (%)	6 (0.7%)	3 (0.5%)	3 (0.8%)	0.685
**Out-patient treatment**, n (%)				
Angiotensin-converting enzyme inhibitors	118 (13.1%)	66 (12.0%)	52 (15.0%)	0.223
Angiotensin II receptor blockers	180 (20.1%)	110 (20.0%)	70 (20.2%)	1.000
Chemotherapy	24 (2.6%)	15 (2.7%)	9 (2.5%)	1.000
Immunosuppressants	24 (2.6%)	16 (2.9%)	8 (2.2%)	0.673
Systemic corticosteroids	47 (5.2%)	27 (4.9%)	20 (5.6%)	0.648
Inhaled corticosteroids	50 (5.5%)	30 (5.4%)	20 (5.6%)	1.000
Acenocumarol	53 (5.8%)	23 (4.2%)	30 (8.4%)	0.009
Low-molecular-weight heparin	27 (3.0%)	22 (4.0%)	5 (1.4%)	0.027
Direct oral anticoagulant	16 (1.7%)	1 (0.2%)	15 (4.1%)	<0.001
Androgen antagonists	8 (0.9%)	1 (0.2%)	7 (1.9%)	0.007
Hydroxychloroquine (HCQ) prior admission	85 (9.3%)	67 (12.1%)	18 (5.0%)	<0.001
Days of HCQ treatment before admission, mean (SD)	4.9 (5.1)	4.1 (3.9)	7.9 (7.7)	0.005
Azithromycin (AZT) prior admission	158 (17.2%)	120 (21.6%)	38 (10.5%)	<0.001
Days of AZT treatment before admission, mean (SD) (n = 158)	4.4 (4.3)	4.2 (4.1)	5.1 (5.1)	0.270
**Symptoms / Signs**				
Duration of symptoms before admission (days) (mean, [SD]) (n = 833)	6.3 (4.9)	6.9 (4.9)	5.4 (4.6)	<0.001
Fever, n (%)	678 (73.9%)	409 (73.7%)	269 (74.1%)	0.939
Duration of fever before admission (days) (mean, [SD])	6.0 (4.6)	6.6 (4.8)	4.9 (4.0)	<0.001
Dry cough, n (%)	438 (47.8%)	280 (50.5%)	158 (43.5%)	0.043
Productive cough, n (%)	116 (12.6%)	61 (11.0%)	55 (15.2%)	0.068
Chest pain, n (%)	82 (8.9%)	63 (11.4%)	19 (5.2%)	0.001
Shortness of breath, n (%)	565 (61.6%)	315 (56.9%)	250 (68.9%)	<0.001
Diminished level of consciousness, n (%)	116 (12.6%)	59 (10.6%)	57 (15.7%)	0.026
Seizures, n (%)	7 (0.8%)	2 (0.4%)	5 (1.4%)	0.120
Fatigue, n (%)	312 (34.0%)	211 (38.1%)	101 (27.8%)	0.001
Myalgia/arthralgia, n (%)	147 (16.0%)	108 (19.5%)	39 (10.7%)	<0.001
Anosmia, n (%)	30 (3.3%)	25 (4.5%)	5 (1.4%)	0.008
Ageusia, n (%)	36 (3.9%)	31 (5.6%)	5 (1.4%)	0.001
Nasal Congestion, n (%)	24 (2.6%)	15 (2.7%)	9 (2.5%)	1.000
Headache, n (%)	49 (5.3%)	40 (7.2%)	9 (2.5%)	0.001
Sore throat, n (%)	0.1 (0.8	0.1 (1.0	0.0 (0.2	0.294
Hemoptysis, n (%)	20 (2.2%)	15 (2.7%)	5 (1.4%)	0.248
Nausea/vomiting, n (%)	104 (11.3%)	82 (14.8%)	22 (6.1%)	<0.001
Abdominal pain, n (%)	44 (4.8%)	33 (6.0%)	11 (3.0%)	0.057
Diarrhea, n (%)	175 (19.1%)	138 (24.9%)	37 (10.2%)	<0.001
Labored breathing, n (%)	332 (36.3%)	132 (23.8%)	200 (55.7%)	<0.001
Conjunctivitis, n (%)	3 (0.3%)	1 (0.2%)	2 (0.6%)	0.566
**Admission measures**				
Temperature, °C, mean (SD) (n = 916)	37.1 (0.9)	37.1 (1.0)	37.0 (0.9)	0.04
Heart rate, beats/min, mean (SD) (n = 917)	87.6 (18.0)	86.6 (16.8)	89.1 (19.7)	0.041
Systolic Blood pressure, mm Hg, mean (SD) (n = 917)	125.9 (23.3)	127.2 (22.7)	123.8 (24.1)	0.032
Dyastolic Blood pressure, mm Hg, mean (SD) (n = 917)	89.7 (15.0)	90.9 (13.7)	87.9 (16.8)	0.003
*Glasgow Coma Scale*,(n = 916)	14.3 (2.1)	14.6 (1.5)	13.8 (2.7)	<0.001
Pulmonary infiltrates on chest x ray, n (%)	841 (91.6%)	499 (89.9%)	342 (94.2%)	0.021
Bilateral pulmonary infiltrate, n (%)	741 (80.7%)	423 (76.2%)	318 (87.6%)	<0.001
Oxygen supplementation, n (%)	455 (49.6%)	214 (38.6%)	241 (66.4%)	<0.001
PaO2 mmHg, mean (SD) (n = 341)	84.3 (19.1)	85.9 (18.0)	82.6 (20.2)	0.107
FiO2%, mean (SD)	31.3 (20.4)	25.6 (10.5)	40.1 (27.6)	<0.001
SpO2%, mean (SD)	91.7 (6.5)	93.3 (4.6)	89.2 (8.1)	<0.001
SpO2/FiO2 ratio, mean (SD)	354.1 (108.9)	391.7 (78.7)	296.3 (123.0)	<0.001

Unless indicated, n = 918 for each variable

**Table 2:** Admission laboratory findings of patients from internal validation cohort by outcome.

		Severity of disease progression		
Characteristics, mean (SD)	Total (n = 918)	Non-severe (n = 555)	Severe (n = 363)	*P*-Value
Hemoglobin, g/dL (n = 911)	13.6 (2.1)	13.9 (2.0)	13.2 (2.2)	<0.001
Reticulocytes (n = 96)	37.8 (20.8)	34.5 (16.9)	44.0 (25.7)	0.031
White blood cells count, x10^9^ /L (n = 910)	8.5 (14.1)	8.4 (17.8)	8.6 (4.2)	0.826
Neutrophil cell count, x10^9^ /L (n = 909)	6.2 (3.6)	5.7 (3.2)	7.1 (4.0)	<0.001
Lymphocyte count, x10^9^ /L (n = 910)	1.7 (12.9)	2.1 (16.5)	1.0 (0.8)	0.188
Monocyte count, x10^9^ /L (n = 910)	0.5 (0.5)	0.5 (0.6)	0.5 (0.4)	0.234
Basophil count, x10^9^ /L (n = 910)	0.0 (0.0)	0.0 (0.0)	0.0 (0.0)	0.010
Platelet-to-lymphocyte ratio, % (n = 910)	262.4 (226.6)	245.0 (238.5)	289.3 (204.1)	0.004
Neutrophil–to-lymphocyte Ratio, % (n = 909)	8.2 (7.9)	6.6 (5.7)	10.6 (10.0)	<0.001
Platelet count x10^9^ /L (n = 912)	209.0 (91.2)	212.2 (92.1)	204.0 (89.8)	0.183
Prothrombin time, % (n = 851)	82.9 (22.0)	85.9 (18.5)	78.3 (25.8)	<0.001
INR (n = 850)	1.4 (2.0)	1.3 (1.6)	1.7 (2.4)	0.010
Activated partial thromboplastin time, s (n = 622)	35.2 (8.3)	35.1 (7.9)	35.4 (8.8)	0.686
Fibrinogen levels, mg/dL (n = 791)	637.3 (200.6)	618.8 (193.9)	666.2 (207.5)	0.001
D-dimer level, μg/mL (n = 846)	2.9 (9.1)	2.5 (9.2)	3.4 (8.9)	0.165
Elevated d-dimer level (n = 846)	465 (49.8%)	252 (47.8%)	213 (64.2)	<0.001
ISTH-DIC score (n = 748)	1.9 (1.2)	1.6 (1.2)	2.3 (1.2)	<0.001
SOFA Score	1.7 (1.9)	1.1 (1.3)	2.7 (2.1)	<0.001
C-reactive protein, mg/dL (n = 903)	13.2 (10.8)	10.6 (9.0)	17.2 (12.1)	<0.001
Creatinine, mg/dL (n = 914)	1.2 (0.7)	1.1 (0.6)	1.5 (0.9)	<0.001
Bilirubin (total), mg/dL (n = 898)	0.6 (0.5)	0.6 (0.5)	0.6 (0.4)	0.439
Ferritin, ng/mL (n = 500)	1237.0 (1449.8)	1052.9 (966.6)	1610.9 (2069.6)	<0.001
Glucose, mg/dL (n = 909)	138.5 (63.8)	130.1 (57.9)	151.5 (70.2)	<0.001
Urea, mg/dL (n = 910)	58.7 (44.2)	45.9 (33.6)	78.4 (51.0)	<0.001
Uric acid, mg/dL (n = 753)	5.4 (2.5)	4.8 (2.1)	6.3 (2.7)	<0.001
eGFR, mL/min/1.73 m² (n = 914)	63.4 (25.6)	71.7 (23.1)	50.7 (23.9)	<0.001
Calcium, mg/dL (n = 849)	8.9 (0.7)	9.0 (0.6)	8.8 (0.7)	<0.001
Magnesium, mmol/L (n = 843)	2.1 (0.4)	2.1 (0.3)	2.2 (0.4)	0.001
Sodium, mmol/L (n = 910)	137.8 (6.8)	137.3 (5.6)	138.7 (8.2)	0.004
Potassium, mmol/L (n = 885)	4.1 (0.6)	4.0 (0.5)	4.2 (0.6)	<0.001
Alanine Aminotransferase, U/L (n = 868)	39.9 (72.5)	38.4 (33.9)	42.2 (108.3)	0.451
Aspartate Aminotransferase, U/L (n = 549)	62.5 (175.7)	49.3 (35.1)	89.0 (299.0)	0.012
Alkaline phosphatase, U/L (n = 865)	83.9 (68.4)	80.8 (60.9)	88.8 (78.6)	0.092
Gamma-glutamyltransferase, *U/L* (n = 866)	72.2 (142.3)	71.6 (108.8)	73.2 (183.0)	0.879
Lactate dehydrogenase, U/L (n = 873)	381.2 (175.1)	345.3 (128.6)	437.5 (218.5)	<0.001
Proteins, g/L (n = 845)	7.5 (0.6)	7.5 (0.6)	7.4 (0.7)	0.001
Albumin, g/L (n = 804)	3.6 (0.4)	3.8 (0.4)	3.5 (0.4)	<0.001
Creatine kinase, U/L (n = 803)	225.9 (504.2)	177.5 (364.4)	304.0 (664.6)	0.001
Procalcitonin, ng/mL (n = 671)	1.2 (6.1)	0.7 (5.4)	1.9 (6.9)	0.018
Bicarbonate, mEq/L (n = 342)	25.2 (5.3)	25.6 (4.7)	24.7 (5.8)	0.101
Base excess, mEq/L (n = 334)	1.0 (5.1)	1.7 (4.3)	0.2 (5.6)	0.006

INR: International normalized ratio; International Society Thrombosis Hemostasis-Disseminated Intravascular Coagulation; SOFA, Sequential Organ Failure Assessment; eGFR, estimated glomerular filtration rate calculated using Chronic Kidney Disease Epidemiology Collaboration (CKD-EPI) equation. Unless indicated, n = 918 for each variable

### Data preparation

The data was preprocessed by one-hot encoding multicategory features and completing missing values with the trimmed mean between 5–95 percentiles and mode of each continuous and categorical feature, respectively.

### Training and validation of the classification machine-learning model

Three machine-learning classifiers typically used in data sets composed by heterogeneous features [7] were trained: random forest [8], xgboost [9] and regularized logistic regression. The development cohort data was split in a train and validation data set following a 10-stratified fold cross-validation scheme with 10 repetitions [10], and the validation results in each of these splits were averaged to assess the performance of the classifiers. In the training phase, all models were optimized by fine tuning their hyperparameters with 10-fold cross-validation scheme and a grid search algorithm, configuring a nested cross-validation scheme to first perform this hyperparameter optimization and secondly internally evaluate the classifier. The fixed values of not optimized hyperparameters and the ranges of optimized ones for each classification and feature selection algorithm can be consulted in S1 Table.

The code to develop the models was written in Python and open source libraries scikit-learn [11], xgboost and eli5 were used for the implementation of the machine-learning classifiers and cross-validation schemes. The code can be consulted at http://github.com/hus-ml/covid19salamanca-score

In order to better assess the clinical significance of our results, a real-world application of the model was evaluated with patients from a second tertiary university center, the Hospital Clinic of Barcelona.

### Evaluation metrics

The differences in clinical, epidemiological and analytical variables between patients with and without severe disease progression at both hospitals were compared using χ2 tests for categorical variables and Student’s t-test for continuous variables.

The performance of the model was evaluated by calculating the area under the receiver-operating-characteristic curve (AUC) and its confidence interval for each prediction model [12, 13]. The classification performance at particular cutoff thresholds based on the receiver-operating-characteristic curve were evaluated according to its sensitivity, specificity, positive predictive value, and negative predictive value.

### Severity of disease classification calculator

The developed machine-learning model was deployed in an open source calculator that can be run on a web application (https://covid19salamanca-score.herokuapp.com), in which Covid-19 patients at hospital admission can be individually stratified as high and non-high risk for severity of disease progression.

To develop a friendly and practical calculator, the number of features used by the machine-learning model was reduced from 140 to less than 10. In order to ensure that all relevant clinical features were present a number of additional models were built using combinations of outcomes (death or death plus mechanical ventilation as labels) and restricting the data set to subgroups (older of 75 years of age, younger than 75 years of age or without age-restriction). The performance of these models were compared and the importance of each feature for these models was computed using Mean Decrease Accuracy [9]. We tallied the number of times each feature appeared as one of the most important in a model and chose the most frequent features. Additionally, correlated features with similar importance were chosen by clinical significance and by their availability in the external data set. We selected a final number of seven features as 8^th^ and 9^th^ variables were of much less importance according to mentioned criteria. The new model developed with the selected features was validated to ensure similar results to the original one with all the features.

## Results

### Development cohort

Between March 1^st^ and April 23^rd^ 2020, among 918 patients that had been admitted at the University Hospital of Salamanca because of SARS-CoV-2 pneumonia, 363 patients (39.5%) died or required mechanical ventilation by May 15th (312 patients died and 82 required mechanical ventilation -31 of them finally died-) and 555 patients (60.5%) did not progress to critical illness and had been discharged by that date. Cause of death was directly related to Covid-19 in 297 patients and to other causes in 15 patients. Diagnosis was confirmed by RT-PCR assay from nasopharyngeal swab or immunochromatography assay in 859 and 59 patients, respectively. Table 1 shows features of patients of this cohort by severity of disease progression.

Concerning clinical variables, patients with severe disease progression were older (average age 79.2 years) and presented with a higher updated Charlson comorbidity index (mean value of 1.8). Overall, patients who developed critical illness had more cardiovascular and central nervous system diseases, and 35 out of 50 patients (70%) with chronic kidney disease had severe disease progression. Cancer was more prevalent in those with severity of disease progression (19.3% vs. 10.8%). Regarding clinical manifestations at admission, shortness of breath and labored breathing were present in 68.9% and 55.7% of the patients who progressed to severe disease, respectively. This group of patients had a significantly lower ratio of oxygen saturation as measured by pulse oximetry divided by the fraction of inspired oxygen (391.7 vs. 296.3) and 66.4% of them required oxygen supplement at admission whilst only 38.6% in the non-severe progression group.

Table 2 represents the laboratory findings at the time of admission by outcome. The patients with severity of disease progression presented at admission with neutrophilia, lymphopenia and higher levels of D-dimer, ferritin, C-reactive protein, procalcitonin and fibrinogen. The critically ill group patients had altered renal function at admission, measured by increased urea and creatinine levels and reduced estimated glomerular filtration rate.

### Risk model performance

**Fig 1:**
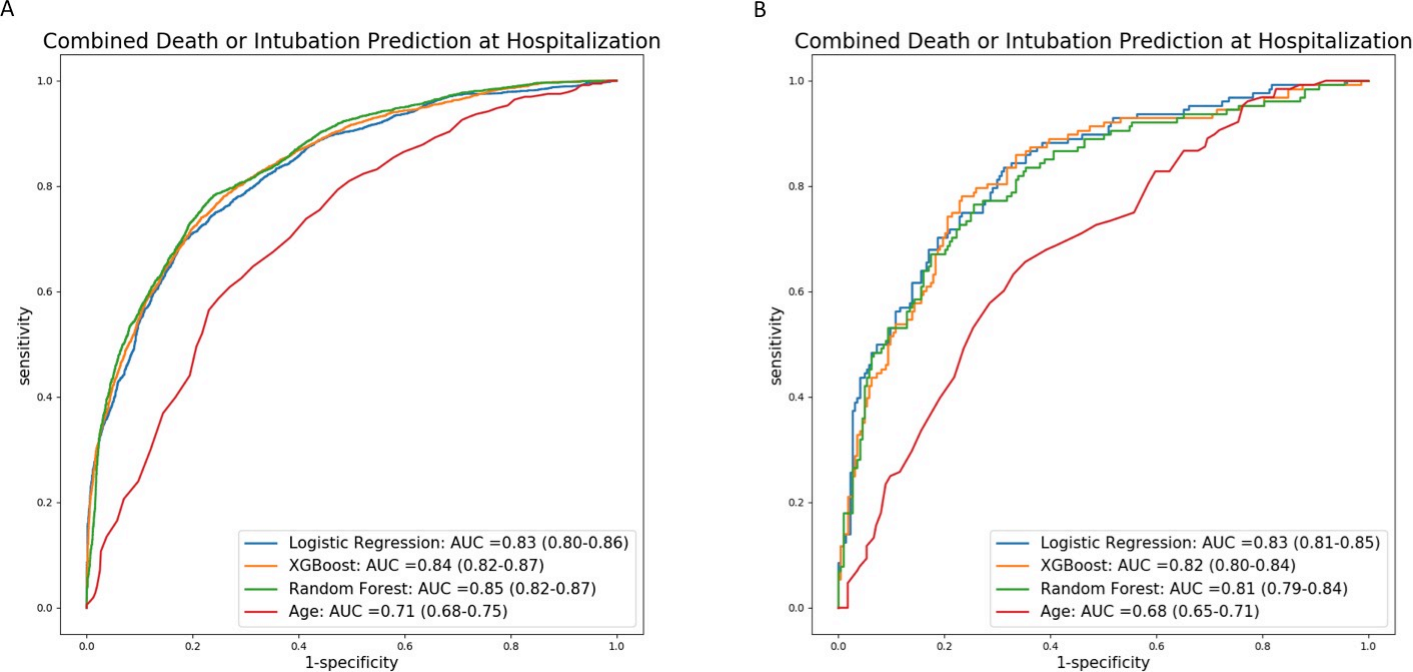
Receiver operating characteristic curves of the machine-learning model for the different classification algorithms. Panel A shows the internal crossvalidation results and panel B shows the external testing results. The results of a risk classification only based on age are also shown for comparison.

In order to develop the risk model, we first selected all variables included in the Tables 1 and 2. Using all the cohort patients and variables, the best model obtained in the internal cross-validation an AUC of 0.86 (CI: 0.83–0.88). With the aim of developing a more user-friendly application and according to the described methodology, we identified 7 variables present in all models with independent prognostic significance: peripheral blood oxygen saturation (SpO2)/fraction of inspired oxygen (FiO2) ratio, age, estimated glomerular filtration rate (calculated using Chronic Kidney Disease Epidemiology Collaboration [CKD-EPI] equation), procalcitonin, C-reactive protein, updated Charlson comorbidity index (detailed in S2 Table) and lymphocytes. By restricting to these 7 variables, out of the 3 trained machine-learning classifiers, the best classifier achieved a highest mean AUC of 0.85 (CI: 0.82–0.87) from our development cohort without significant difference among them (Fig 1).

### External testing cohort

Between February 15^th^ and April 28^th^ 2020, 352 patients were admitted at the Clinic Hospital of Barcelona because of their first episode of SARS-CoV-2 pneumonia confirmed by RT-PCR assay from nasopharyngeal swab. Among them, 128 (36.3%) patients developed critical illness (64 died and 77 required mechanical ventilation -13 of them finally died-) and 224 (63.6%) did not and were discharged by May 20th. Cause of death was directly attributed to Covid-19 in all patients but three. The baseline characteristics and laboratory findings at the time of admission in this external testing cohort are represented in the Table 3. Patients with severity of disease progression were older (median age of 68.7), with lower SpO2/FiO2%, lower glomerular filtration rate and higher procalcitonin and C-reactive protein values. In addition, they presented with lower lymphopenia count and higher updated Charlson Comorbidity index scores.

**Table 3:** Admission demographic and clinical characteristics of patients from external testing cohort by outcome.

		Severity of disease progression		
Characteristics	Total (n = 352)	No (n = 224)	Yes (n = 128)	*P*-value
Male, n (%)	235 (66.8%)	148 (66.1%)	87 (68.0%)	0.726
Age, mean (SD)	62.9 (14.6)	59.5 (15.1)	68.7 (11.6)	<0.001
SpO2/FiO2, %/%, mean (SD)	3.80 (1.09)	4.18 (0.69)	3.11 (1.32)	<0.001
eGFR, mL/min/1.73 m², mean (SD)	77.6 (24.4)	83.0 (21.6)	67.9 (26.3)	<0.001
Procalcitonin, ng/mL, mean (SD)	0.6 (2.7)	0.3 (0.8)	1.1 (4.3)	0.035
Lymphocyte count, x10^9^ /L, mean (SD)	0.8 (0.4)	0.9 (0.4)	0.7 (0.4)	<0.001
C-reactive protein, mg/L, mean (SD)	11.9 (14.7)	8.6 (6.9)	17.9 (21.7)	<0.001
Updated Charlson comorbidity index, mean (SD)	0.8 (1.0)	0.6 (0.9)	1.1 (1.1)	<0.001

FiO2, fraction of inspired oxygen; SpO2, arterial oxygen saturation measured by pulse oximetry; eGFR, estimated glomerular filtration rate.

The three trained classifiers restricted to the 7 most relevant variables were externally validated on this cohort. In this case, the best classifier obtained a mean AUC of 0.83 (CI: 0.81–0.85), again without significant differences respect to the other classifiers (Fig 1) and very consistent with the results obtained in the development cohort.

The relative contribution to the AUC of each feature both in the development and testing populations are shown in Table 4. In both cohorts, SpO2/FiO2 and C-reactive protein were the best predictors of critical evolution of disease, while procalcitonin and lymphocyte count showed lower contribution to the prediction.

**Table 4:** Relative importance of each variable according to mean decrease accuracy, scaled to the most important one.

	Logistic Regression	Random Forest	XGBoost
SpO2/FiO2	1	1	1
C-reactive protein	0.353	0.218	0.102
eGFR	0.381	0.328	0.200
Age	0.26	0.291	0.150
Updated Charlson comorbidity index	0.19	0.185	0.076
Lymphocyte count	0.039	0.183	0.097
Procalcitonin	0.003	0.213	0.179

SpO2, arterial oxygen saturation measured by pulse oximetry; FiO2, fraction of inspired oxygen; eGFR, estimated glomerular filtration rate

### Calculator application

The 7-variable model based on the regularized logistic regression, which obtained the best result in the external testing cohort, has been deployed in an open-source web calculator (https://covid19salamanca-score.herokuapp.com/) to predict the risk of severity of disease progression, with the possibility of selecting different cut-off thresholds according to the desired sensitivity for the detection of high-risk patients. This also allows to individualize this threshold depending on the availability of hospital resources (e.g., higher resources may allow more sensitivity to detect high-risk patients). As an example, we have predefined a high availability resource cut-off threshold, which is estimated to obtain in the internal validation cohort a sensitivity of 0.90 and specificity of 0.52 for detecting high-risk patients. This threshold results in the identification of 2 groups of patients representing the 64.6% and 35.4% of the cohort with 55.1% and 11.2% of them developing severe disease, respectively (S2 Fig). These values of sensitivity and specificity are susceptible to change in populations with different risk distributions (e.g., younger populations) or if there are other pre-admission criteria that skew the population. As a consequence, this high availability resource cut-off threshold, evaluated on the external setting cohort (younger population), identified groups including the 39.2% and 60.8% of the population with 65.9% and 17.3% of them developing severe disease, respectively. S3 Table shows the values of sensibility, specificity, precision, and negative predictive value for these two possible thresholds (high and low resource availability).

## Discussion

In this study, we have developed and validated, through machine-learning, a clinical risk score to predict at the moment of hospital admission by Covid-19, the risk of mechanical ventilation or death. This score is also provided as an open-source web-based calculator, which allows clinicians to estimate an individual Covid-19 patient risk and make decisions based on availability of resources for critical patients and patient overload.

This score includes several common and readily available variables that may be collected at admission in most hospitals. Both development and testing cohorts of patients are representative series for gaining insights into the prediction of disease severity in Covid-19 patients because both are university institutions, patients were in charge of Infectious Diseases/Internal Medicine Departments and treatment protocols were quite homogeneous due to the recommendations of the Spanish Agency of Medicines and Medical Devices (AEMPS). Further, the selected time frame corresponds to the peak Covid-19 incidence and excess mortality in Spain.

As far as the variables included in the risk model here presented, age has been described as one of the main risk factors predicting severity and inpatient mortality in Covid-19 and other scores have also included this variable [2, 14]. Concerning comorbidities, although the exact type and number of comorbidities posing more risk for adverse outcomes is still unknown, our analysis has shown that updated Charlson comorbidity index was the most powerful variable to integrate and combine comorbidities at admission and resulted better than individual variables, such as hypertension or heart failure, or the classical Charlson index. Considering that the updated Charlson index is an improved and more parsimonious prognostic score than the classical one, has been previously shown to be a useful tool to reduce potential confounding in epidemiological research, and has been described as a prognostic tool in many settings, including infectious diseases [15, 16], this score may therefore serve to adjust for comorbidity in other Covid-19 studies.

The ratio of oxygen saturation as measured by pulse oximetry divided by the fraction of inspired oxygen is a simple measure, which has been previously used in the setting of acute respiratory distress syndrome instead of more complex variables [17], and thus can be evaluated in each patient with Covid-19 pneumonia to help identify patients at higher risk of severe disease.

Regarding laboratory variables, decreased estimated glomerular filtration rate and increased acute phase reactants like procalcitonin or C-reactive protein are associated with higher risk of severe disease. Although renal disease is part of the Charlson index as a comorbid disease, decreased estimated glomerular filtration rate may indicate not only the presence of this comorbidity but also acute kidney injury due to disease severity (e.g., septic shock). Therefore, it is a simple variable to assess severity of disease progression at the time of first visit. Indeed, kidney disease as a predictor of increased Covid-19 inpatient mortality rate has been previously described in a single-center study in China [18].

Increased levels of C-reactive protein and its association with prognosis and severity in Covid-19 have been reported and correlated with pro-inflammatory response [19]. Disease severity has also been linked with increased procalcitonin levels and described in some series although its elevation might be likely associated with the presence of bacterial superinfection [20]. Low lymphocyte count has already been linked to poorer outcomes in Covid-19 inpatients and other viral infections such as influenza [19, 21]. In addition, lymphopenia may play a pathogenic role in this disease due to a decrease of specific lymphocyte subpopulations and tissue infiltration [22].

Machine-learning models incorporate classical methods such as multivariate logistic regression but also add regularization terms and cross validation schemes, which makes the models more robust against overfitting and allows more prediction accuracy for each variable. The score presented here exhibits a very good performance and accuracy, as well as excellent validation in the testing cohort with an easy-to-use web interface. It is of note that our results are also quite consistent with a recent study from Spain which identified advanced age, several comorbidities included in the Charlson index, age-adjusted oxygen saturation, higher concentrations of C-reactive protein, and lower estimated glomerular filtration rate as independent factors associated with increased hazard of death [23]. Although previous scores for Covid-19 risk prediction have been developed and validated in Chinese [14, 19], European [24, 25] or North American patients [26], our score offers an open-source web calculator based in machine learning methodology, shows a very good AUC value with excellent replication in a testing cohort from another center and uses easy-to-collect variables on admission. For instance, our score offers better AUC (development cohort: 0.85, 95% CI 0.82 to 0.87; validation cohort: 0.83, 0.81 to 0.85) than the 4C mortality score [24] developed in European population (development cohort: 0.79, 95% CI 0.78 to 0.79; validation cohort: 0.77, 0.76 to 0.77). As a potential limitation, we have to acknowledge that elderly patients with several comorbidities may have not been candidates for certain therapies such as invasive or non-invasive mechanical ventilation, which may influence mortality in this subgroup of patients and limit the generalization of our findings. However, the results of our score were very similar between development and external testing cohorts and both centers followed the same recommendations for intensive care treatment during COVID-19 pandemic [27]. In any case, additional validation outside of Spain is needed to ensure generalizability. We would also like to highlight the possibility of the open-source web calculator to select different sensitivity cut-off thresholds to classify patients depending on health-care resources and population risk distributions. This possibility may improve the efficiency of triage of Covid-19 patients at hospital admission through a real-time, automated and personalized method that would also take into account hospital intensive care unit availability within this pandemic situation. Thus, patients with a high risk of severe disease could be early transferred to tertiary care hospitals or to intermediate care units if available in order to close monitoring and early access to non-invasive or invasive mechanical ventilation.

This study, to assure uniformity, only focused on patients admitted to a university hospital after an emergency department visit and did not include those Covid-19 cases managed in the outpatient setting. However, it would be optimal to validate this risk score at the time of first evaluation by family physicians to potentially identify patients at risk of progressive disease and thus allow early hospital referral.

In summary, this risk model may represent a reliable system that uses widely available clinical and laboratory parameters at hospital admission. The application of machine-learning methods has led to better prediction of the outcome for the identification of Covid-19 inpatients that will likely develop progressive disease after admission.

## Supporting information

S1 Dataset (XLS)

S1 Table List of adjusted hyperparameters of each classifier, their tested values and optimized value. (DOCX)

S2 Table Definition and weights of comorbid conditions included in the updated Charlson comorbidity index. (DOCX)

S3 Table Evaluation metrics as measured in the internal validation dataset for the suggested thresholds in the calculator. (DOCX)

S1 Fig Flow chart illustrating steps involved in prognostic score development. (TIF)

S2 Fig Caption of web based calculator. A high availability resource cut-off threshold is estimated to obtain in the internal validation cohort a sensitivity of 0.90 and specificity of 0.52 for detecting high-risk patients. Web based calculator available at https://covid19salamanca-score.herokuapp.com/.
(TIF)
