# Correction: Development of a severity of disease score and classification model by machine learning for hospitalized COVID-19 patients

**DOI:** 10.1371/journal.pone.0315526

**Published:** 2024-12-05

**Authors:** Miguel Marcos, Moncef Belhassen-García, Antonio Sánchez-Puente, Jesús Sampedro-Gomez, Raúl Azibeiro, Pedro-Ignacio Dorado-Díaz, Edgar Marcano-Millán, Carolina García-Vidal, María-Teresa Moreiro-Barroso, Noelia Cubino-Bóveda, María-Luisa Pérez-García, Beatriz Rodríguez-Alonso, Daniel Encinas-Sánchez, Sonia Peña-Balbuena, Eduardo Sobejano-Fuertes, Sandra Inés, Cristina Carbonell, Miriam López-Parra, Fernanda Andrade-Meira, Amparo López-Bernús, Catalina Lorenzo, Adela Carpio, David Polo-San-Ricardo, Miguel-Vicente Sánchez-Hernández, Rafael Borrás, Víctor Sagredo-Meneses, Pedro-Luis Sanchez, Alex Soriano, José-Ángel Martín-Oterino

There are errors in the Funding statement. The correct Funding statement is as follows: This work was partially supported by Instituto de Salud Carlos III, Ministerio de Ciencia e Innovación (Madrid, Spain) and co-funded by the European Union and FEDER Funds “Una manera de hacer Europa,” by grants CIBERCV CB16/11/00374 to Pedro-Luis Sánchez, PI18/01061 to Carolina Garcia-Vidal, and RD16/0017/0023 to Miguel Marcos, and by Institute of Biomedical Research of Salamanca (IBSAL) through a special grant for Covid-19 research.
