# Supplementary material for: Development of a severity of disease score and classification model by machine learning for hospitalized COVID-19 patients
Source: PLoS One. 2021 Apr 21;16(4):e0240200. doi: 10.1371/journal.pone.0240200 (PMC8059804; doi:10.1371/journal.pone.0240200)
Supplement: S1 Table — (DOCX) [file pone.0240200.s002.docx]

**S1 Table. List of adjusted hyperparameters of each classifier, their tested values and optimized value.**

|  |  |  |  |
| --- | --- | --- | --- |
|  | **Hyperparameter** | **Possible values** | **Best value in grid search** |
| **Logistic Regression** | penalty type | l1,l2,elasticnet | l2 |
|  | penalty coefficient | 0.1,1,10,100 | 0.1 |
| **Random Forest** | tree depth | 1,2,5,unlimited | 5 |
|  | max features | 1, sqrt(n) | 1 |
|  | estimators | 100 | 100 |
| **XGBoost** | gamma | 0,1,5,20 | 1 |
|  | learning rate | 0.05,0.1,0.25,0.5 | 0.05 |
|  | max depth | 1,2,5 | 2 |
|  | estimators | 100 | 100 |
