# Supplementary material for: Development of a severity of disease score and classification model by machine learning for hospitalized COVID-19 patients
Source: PLoS One. 2021 Apr 21;16(4):e0240200. doi: 10.1371/journal.pone.0240200 (PMC8059804; doi:10.1371/journal.pone.0240200)
Supplement: S2 Table — (DOCX) [file pone.0240200.s003.docx]

**S2 Table. Definition and weights of comorbid conditions included in the updated Charlson comorbidity index**

|  | |  |
| --- | --- | --- |
| Condition | Definition | Weight |
| Congestive heart failure | Patients who have had exertional or paroxysmal nocturnal dyspnea and who have responded symptomatically (or on physical examination) to digitals, diuretics, or afterload reducing agents. It does not include patients who are on medication but have had no symptomatic response and no evidence of improvement of physical signs. | 2 |
| Dementia | Patients with chronic cognitive deficit. | 1 |
| Chronic pulmonary disease | Patients who are dyspneic with slight activity, with or without treatment and those who are dyspneic with moderate activity despite treatment. Also includes patients who are dyspneic at rest, despite treatment, those who require constant oxygen, those with CO_2_ retention and those with baseline PO_2_ below 50 torr. | 1 |
| Rheumatologic disease | Patients with systemic lupus erythematous, polymyositis, mixed connective tissue disease, polymyalgia reumática, and moderate to severe rheumatoid arthritis | 1 |
| Peptic ulcer disease | Patients who have required treatment for ulcer disease, including those who have bled from ulcers |  |
| Mild liver disease | Patients with chronic hepatitis or cirrhosis without portal hypertension. | 2 |
| Diabetes with chronic complication | Patients with retinopathy, neuropathy, or nephropaty. | 1 |
| Hemiplegia or paraplegia | Patients with the dense hemiplegia or paraplegia, whetever it occurred as a result of a cerebrovascular accident or other condition. | 22 |
| Renal disease | Patients with serum creatinine > 2 mg% or patients on dialysis, those who had a transplant, and those with uremia. | 1 |
| Any malignancy without metastasis | Patients with solid tumors without documented metastases, but initially treated in the last five years, including breast, colon, lung and a variety of other tumors (includes leukemia and lymphoma) | 2 |
| Moderate or severe liver disease | Patients with cirrohsis with portal hypertension with or whithout a history of variceal bleeding. | 4 |
| Metastatic solid tumour | Patients with metastátic solid tumors, including breast, lung, colon and other tumors. | 6 |
| Acquired immune deficiency syndrome | Patients with define or probable acquired immune deficiency syndrome. Patients with only human immune defiency virus infection but asymptomatic were not designated as having acquired immune deficiency syndrome. | 4 |

  To calculate the index the following comorbid conditions were mutually exclusive: mild liver disease and moderate or severe liver disease; and any malignancy and metastatic solid tumour.
