# Supplementary material for: Development of a severity of disease score and classification model by machine learning for hospitalized COVID-19 patients
Source: PLoS One. 2021 Apr 21;16(4):e0240200. doi: 10.1371/journal.pone.0240200 (PMC8059804; doi:10.1371/journal.pone.0240200)
Supplement: S3 Table — (DOCX) [file pone.0240200.s004.docx]

**S3 Table.** Evaluation metrics as measured in the internal validation dataset for the suggested thresholds in the calculator

|  | TP | FP | TN | FN | R | S | P | NPV |
| --- | --- | --- | --- | --- | --- | --- | --- | --- |
| High Resource Availability Suggested Threshold | 35.6% | 28.7% | 31.7% | 4.0% | 90.0% | 52.5% | 55.3% | 88.9% |
| Limited Resource Availability Suggested Threshold | 21.1% | 5.9% | 54.6% | 18.5% | 53.3% | 90.3% | 78.2% | 74.7% |

FP= false positive; FN= false negative; NPV= negative predictive value; P=precision; R= recall or sensibility; S= specificity; TP= true positive; TN= true negative
