# Supplementary figures and images for: Development of a severity of disease score and classification model by machine learning for hospitalized COVID-19 patients
Source: PLoS One. 2021 Apr 21;16(4):e0240200. doi: 10.1371/journal.pone.0240200 (PMC8059804; doi:10.1371/journal.pone.0240200)

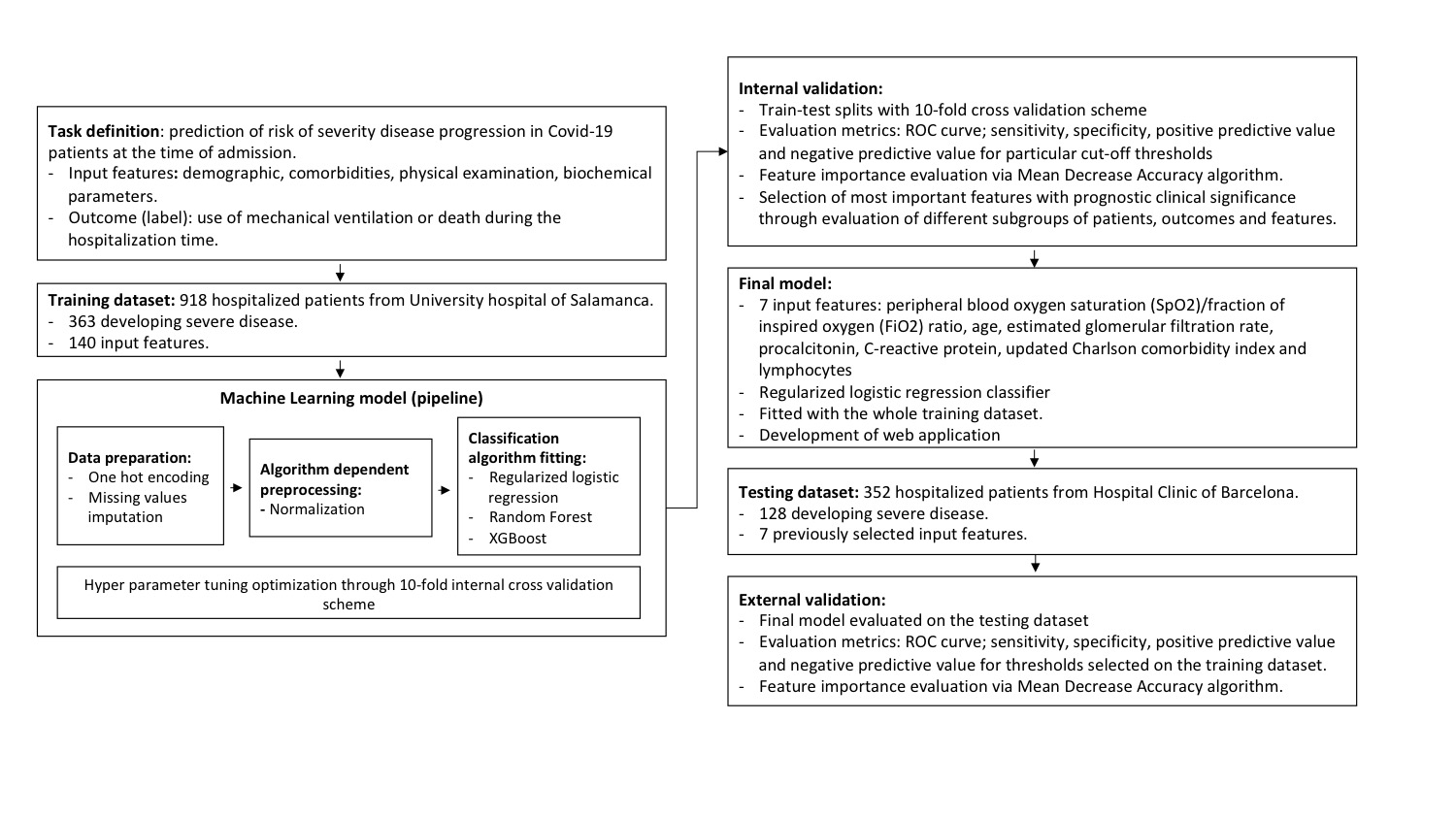

Supplement: S1 Fig — (TIF) [file pone.0240200.s005.tif]

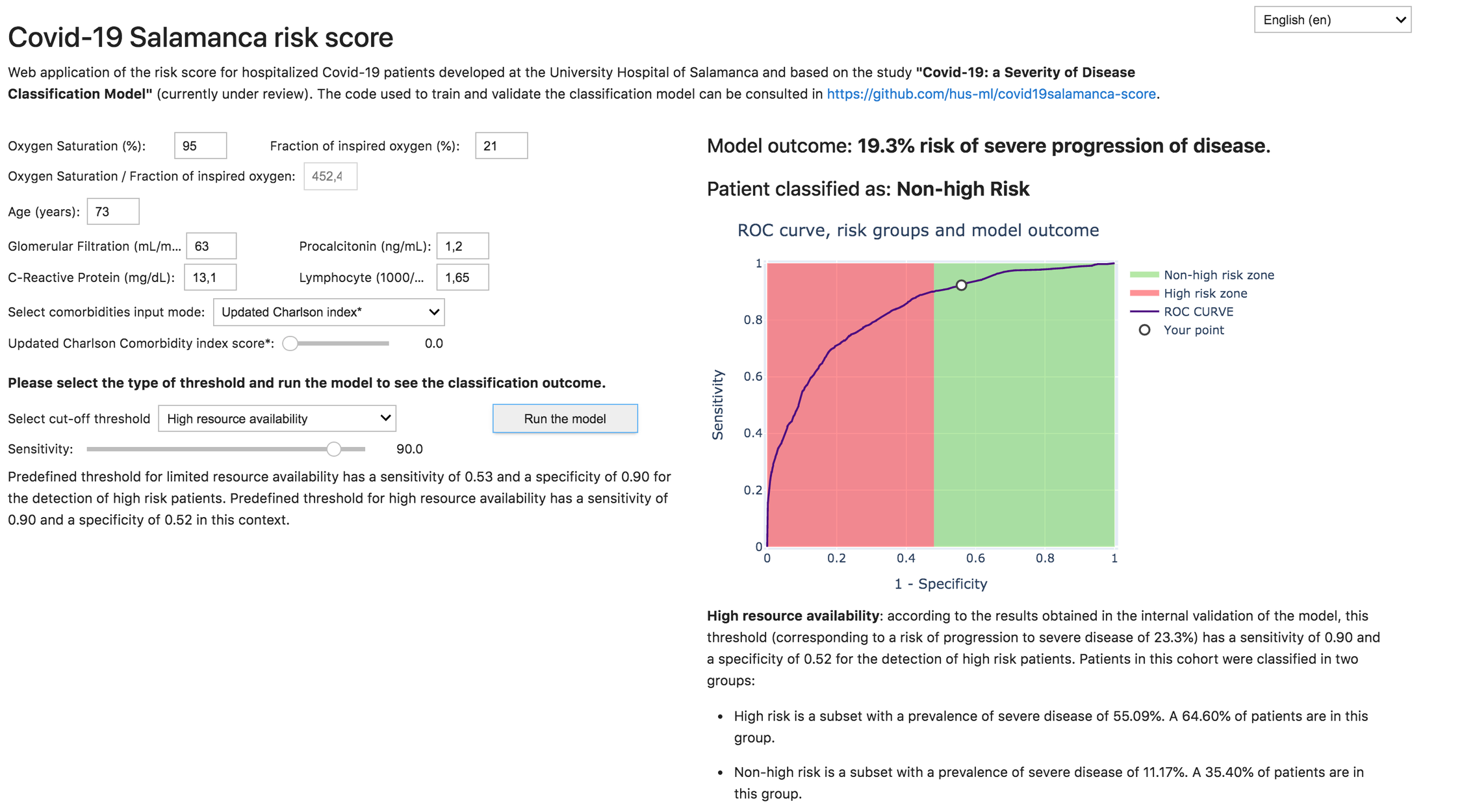

Supplement: S2 Fig — A high availability resource cut-off threshold is estimated to obtain in the internal validation cohort a sensitivity of 0.90 and specificity of 0.52 for detecting high-risk patients. Web based calculator available at https://covid19salamanca-score.herokuapp.com/. (TIF) [file pone.0240200.s006.tif]
